# Development of a Cell-Based High-Throughput Screening Assay to Identify Porcine Host Defense Peptide-Inducing Compounds

**DOI:** 10.1155/2018/5492941

**Published:** 2018-11-19

**Authors:** Zhuo Deng, Jing Wang, Wentao Lyu, Xuwen Wieneke, Robert Matts, Xi Ma, Guolong Zhang

**Affiliations:** ^1^Department of Animal and Food Sciences, Oklahoma State University, Stillwater, OK 74078, USA; ^2^Institute of Animal Husbandry and Veterinary Medicine, Beijing Academy of Agricultural and Forestry Sciences, Beijing 100097, China; ^3^Department of Biochemistry and Molecular Biology, Oklahoma State University, Stillwater, OK 74078, USA; ^4^State Key Laboratory of Animal Nutrition, China Agricultural University, Beijing 100193, China; ^5^Department of Physiological Sciences, Center for Veterinary Health Sciences, Oklahoma State University, Stillwater, OK 74078, USA

## Abstract

Novel alternatives to antibiotics are needed for the swine industry, given increasing restrictions on subtherapeutic use of antibiotics. Augmenting the synthesis of endogenous host defense peptides (HDPs) has emerged as a promising antibiotic-alternative approach to disease control and prevention. To facilitate the identification of HDP inducers for swine use, we developed a stable luciferase reporter cell line, IPEC-J2/*PBD3-luc*, through permanent integration of a luciferase reporter gene driven by a 1.1 kb porcine *β*-defensin 3 (*PBD3*) gene promoter in porcine IPEC-J2 intestinal epithelial cells. Such a stable reporter cell line was employed in a high-throughput screening of 148 epigenetic compounds and 584 natural products, resulting in the identification of 41 unique hits with a minimum strictly standardized mean difference (SSMD) value of 3.0. Among them, 13 compounds were further confirmed to give at least a 5-fold increase in the luciferase activity in the stable reporter cell line, with 12 being histone deacetylase (HDAC) inhibitors. Eight compounds were subsequently observed to be comparable to sodium butyrate in inducing *PBD3* mRNA expression in parental IPEC-J2 cells in the low micromolar range. Six HDAC inhibitors including suberoylanilide hydroxamine (SAHA), HC toxin, apicidin, panobinostat, SB939, and LAQ824 were additionally found to be highly effective HDP inducers in a porcine 3D4/31 macrophage cell line. Besides *PBD3*, other HDP genes such as *PBD2* and cathelicidins (*PG1–5*) were concentration-dependently induced by those compounds in both IPEC-J2 and 3D4/31 cells. Furthermore, the antibacterial activities of 3D4/31 cells were augmented following 24 h exposure to HDAC inhibitors. In conclusion, a cell-based high-throughput screening assay was developed for the discovery of porcine HDP inducers, and newly identified HDP-inducing compounds may have potential to be developed as alternatives to antibiotics for applications in swine and possibly other animal species.

## 1. Introduction

Subtherapeutic antibiotics are commonly used in the swine industry for growth promotion and disease control and prevention. However, due to a rise in antimicrobial resistance, such a practice has been largely phased out in the US. Novel approaches to disease intervention are needed. As a major component of the innate immune system, host defense peptides (HDPs) are broadly active against Gram-positive and Gram-negative bacteria, viruses, and fungi [1–3]. HDPs are also capable of controlling and limiting infections by recruiting different types of immune cells to the site of infection and further neutralizing endotoxin-induced inflammation [1–3]. A few HDPs were recently found to have barrier protective properties [4]. HDPs and their mimetics are, therefore, being actively explored as vaccine adjuvants or novel antimicrobials to treat resistant infections [1–3].

Cathelicidins and defensins constitute two major families of HDPs in mammals. The human genome harbors one cathelicidin (LL-37), six *α*-defensins, and more than 30 *β*-defensins [5]. Approximately a dozen cathelicidins such as protegrins (PG1–5) and 29 porcine *β*-defensins (PBDs) have been reported in pigs [6, 7]. As an important first line of host defense, HDPs are mainly produced in phagocytic leukocytes as well as in mucosal epithelial cells on the surface of gastrointestinal, respiratory, and urogenital tracts. Besides infection and inflammation, a number of small-molecule compounds such as butyrate, vitamin D_3_, isoleucine, and bile acids were recently found to induce HDP genes in humans and other animals [8–10]. In fact, augmenting the synthesis of endogenous HDPs has been found to enhance bacterial clearance and could be explored as a novel approach to host-directed antimicrobial therapy [8–10].

Butyrate together with other short- and medium-chain fatty acids is capable of inducing HDP synthesis not only in humans but also in rabbits, cattle, chickens, and pigs [11–20]. For example, multiple porcine HDP genes such as *PBD2*, *PBD3*, and *PG1–5* have been shown to be induced by a number of fatty acids including butyrate in porcine IPEC-J2 intestinal epithelial cells, 3D4/31 lung alveolar macrophage cells, and primary monocyte cells [20, 21]. However, a few other HDP inducers such as vitamin D_3_ are strong HDP inducers in humans [22, 23] but are weaker in chickens and cattle [24–26]. Vitamin D_3_ fails completely to induce cathelicidin gene expression in mice due to a lack of the vitamin D receptor in the mouse cathelicidin gene promoter, suggesting the existence of species-specific regulation of HDP genes [27]. To identify potent HDP inducers specific for swine applications, we developed a cell-based high-throughput screening (HTS) assay in this study and identified multiple small-molecule compounds that could be potentially developed as alternatives to antibiotics for swine disease control and prevention.

## 2. Materials and Methods

### 2.1. Cell Culture

A porcine intestinal epithelial cell line, IPEC-J2 [28], was cultured in Dulbecco's modified Eagle medium (DMEM)/Ham's F12 medium (Invitrogen, Carlsbad, CA) supplemented with 10% fetal bovine serum (FBS) (Atlanta Biologicals, Lawrenceville, GA), 1% ITS premix (5 *μ*g/ml insulin, 5 *μ*g/ml transferrin, and 5 ng/ml selenium) (Sigma-Aldrich, St. Louis, MO), 5 ng/ml epidermal growth factor (Sigma-Aldrich, St. Louis, MO), 100 U/ml penicillin, and 100 *μ*g/ml streptomycin (Invitrogen). A porcine lung alveolar macrophage cell line, 3D4/31 [29], was cultured in Roswell Park Memorial Institute (RPMI) 1640 medium (Lonza, Allendale, NJ) supplemented with 10% FBS, 100 U/ml penicillin, and 100 *μ*g/ml streptomycin. Both cell types were maintained at 37°C and 5% CO_2_.

### 2.2. Cloning of a Porcine *PBD3* Gene Promoter-Driven Luciferase Reporter Vector

Porcine genomic DNA was isolated from a segment of the jejunum using the Quick-gDNA MiniPrep Kit (Zymo Research, Irvine, CA) and subsequently used as the template for PCR amplification of a 1121 bp promoter fragment using the Advantage 2 PCR Kit (Clontech, Mountain View, CA). The forward and reverse primers are 5′-TGG CCT AAC TGG CCG GTA CCT GAA CTG CCC CTC TTT GCA TCT-3′ and 5′-CCG GAT TGC CAA GCT TTA AAG ATT CCA GGT CCA CAG CCA-3′, respectively, where gene-specific sequences are located at the 3′-regions and underlined sequences are included for In-Fusion PCR Cloning (Takara Bio USA, Mountain View, CA) as recommended by the manufacturer. It is noted that the 5′-end of the gene-specific reverse primer was designed immediately upstream of the porcine *PBD3* mRNA reference sequence (GenBank accession number NM_214444), and the entire *PBD3* gene promoter fragment is located at chr15: 38,063,074–38,064,194 of the UCSC Genome Browser assembly ID susScr11. The PCR product was then cloned into a pGL4.21[*luc2P*/Puro] luciferase reporter vector (Promega, Madison, WI) predigested with *Kpn*I and *Hind*III (Promega) using the In-Fusion HD Cloning Kit (Takara Bio USA) following the manufacturer's instructions. Recombinant plasmids were confirmed for the presence of a gene-specific insert by Sanger sequencing.

### 2.3. Construction of a Stable Luciferase Reporter Cell Line

Recombinant reporter plasmid was linearized with *Kpn*I and transfected into IPEC-J2 cells using FuGene HD (Promega) by following the manufacturer's recommendations. In brief, 2 × 10^5^ IPEC-J2 cells/well were seeded in a 6-well plate in 2 ml complete DMEM/F12 medium and incubated overnight, followed by transfection of 750 ng of the linearized plasmid with 2.25 *μ*l FuGene HD reagent. After 48 h incubation, cells were replenished every 2–3 days with fresh complete DMEM/F12 medium containing 1 *μ*g/ml puromycin. After a week of puromycin selection, cells in each well were expanded to a 10 cm cell culture plate for another week. A stable puromycin-resistant luciferase reporter cell line, named IPEC-J2/*PBD3*-*luc*, was maintained in complete DMEM/F12 medium containing 1 *μ*g/ml puromycin for another week and subcultured every 3–4 days, before being used in the HTS assay development.

### 2.4. Development and Optimization of a High-Throughput Screening (HTS) Assay

The responsiveness of the stable IPEC-J2/*PBD3*-*luc* reporter cell line to butyrate was first evaluated by seeding 2 × 10^4^ cells/well in 50 *μ*l of complete DMEM/F12 medium in a 96-well tissue culture plate overnight prior to stimulation with or without different concentrations (4, 8, 16, 32, and 64 mM) of sodium butyrate (Sigma-Aldrich, St. Louis, MO) for 24 h. The luciferase activity was measured with Modulus Luminometer (Turner BioSystems, Sunnyvale, CA) using the Steady-Glo Luciferase Assay System (Promega, Madison, WI) as instructed by the manufacturer. To further assess the robustness of the HTS assay, the *Z*′-factor [30] was calculated by treating the stable reporter cells in a 96-well plate with or without 16 mM butyrate for 24 h, followed by the luciferase assay.

### 2.5. HTS Assay for HDP-Inducing Compounds

An Epigenetics Screening Library was purchased from Cayman Chemical (Ann Arbor, MI) and consists of 148 small-molecule compounds that are involved in different epigenetic modifications such as DNA/histone methylation and demethylation as well as histone acetylation and deacetylation. A natural product library containing 502 compounds and a rare natural product library containing 82 compounds were previously procured from BIOMOL International (Plymouth Meeting, PA) [31]. The compounds in the Epigenetics Screening Library were provided as 10 mM stocks in DMSO, while those in natural product libraries were provided at 1 mg/well and reconstituted in DMSO to 10 mg/ml stocks. The HTS assays were conducted in 96-well plates by seeding the stable IPEC-J2/*PBD3*-*luc* cells at 2 × 10^4^/well. After overnight culture, the cells were stimulated for 24 h with individual compounds at the final concentration of 20 *μ*M for the Epigenetics Screening Library or 20 *μ*g/ml for the natural product libraries. The luciferase activity was measured with an L-Max II Luminescence Microplate Reader (Molecular Devices, Sunnyvale, CA). To assess relative toxicity of each compound, 1/50 volume of alamarBlue Reagent (Invitrogen, Carlsbad, CA) was added to each well 4 h prior to the luciferase assay. The fluorescence was read in live cells at 545 nm excitation and 590 nm emission using an FLx80 Microplate Fluorescence Reader (BioTek Instruments, Winooski, VT). The relative luciferase activity of each compound was normalized to the cell viability to calculate the strictly standardized mean difference (SSMD) for hit selection as described [32].

### 2.6. Validation of Hit Compounds

The compounds with a normalized SSMD value of no less than 3.0 were further assayed for their luciferase activity in the stable IPEC-J2/*PBD3*-*luc* cell line at three different concentrations in 96-well plates. After normalization to the cell viability, the fold change in the luciferase activity of each compound relative to the nonstimulation control was calculated. Compounds showing a minimum 5-fold increase at any of the three concentrations examined were further evaluated in the parental IPEC-J2 cell line and a porcine lung alveolar macrophage cell line 3D4/31 for their ability to induce the mRNA expression of porcine HDP genes. All individual compounds were purchased from Cayman Chemical (Ann Arbor, MI), except for (−)-depudecin, which was procured from BioVision (Milpitas, CA) and MyBioSource (San Diego, CA). Three different concentrations of each compound were applied to the cells in 12-well plates for 24 h, followed by total RNA isolation, reverse transcription, and real-time PCR as described below. All treatments were performed in duplicate and repeated 2–4 times.

### 2.7. RNA Isolation, Reverse Transcription, and Real-Time PCR

After stimulation, cells were directly lysed in RNAzol® RT Reagent (Molecular Research Center, Cincinnati, OH), followed by total RNA isolation as recommended by the manufacturer. The first-strand cDNA was synthesized with 0.3 *μ*g of total RNA in 4 *μ*l reactions using the iScript cDNA Synthesis Kit (Bio-Rad Laboratories, Hercules, CA). Real-time PCR was conducted in 10 *μ*l reactions on the iQ5 Real-Time PCR Detection System (Bio-Rad Laboratories) using the Maxima SYBR Green qPCR Master Mix (Thermo Fisher Scientific, Waltham, MA) according to the manufacturer's instruction. The expression levels of porcine HDP genes including *PBD2*, *PBD3*, and *PG1–5* as well as a housekeeping gene, glyceraldehyde-3-phosphate dehydrogenase (*GAPDH*), were evaluated using gene-specific primers as described [20], and relative fold changes in gene expression were calculated using the ΔΔCt method [17, 20].

### 2.8. Minimum Inhibitory Concentrations of HDAC Inhibitors

The minimum inhibitory concentrations of the selected HDAC inhibitors were determined by a standard broth microdilution assay as recommended by the Clinical and Laboratory Standards Institute [33] as we previously described [34–36]. Briefly, *Escherichia coli* (ATCC 25922) and *Staphylococcus aureus* (ATCC 43300) were cultured in trypticase soy broth (TSB) with shaking at 250 rpm at 37°C for 3 h to reach the midlog phase of growth. Bacteria were then washed twice in 10 mM sodium phosphate buffer (pH 7.4) and diluted to 5 × 10^5^ CFU/ml in Mueller Hinton Broth (MHB, Fisher Scientific). After dispensing 90 *μ*l/well in a 96-well tissue culture plate, 10 *μ*l of SAHA, apicidin, and HC toxin was added in duplicate to final concentrations of 5, 10, 20, 40, 80, 160, and 320 *μ*M. The MIC value of each compound was determined as the lowest concentration of the compound that gave no visible bacterial growth after overnight incubation at 37°C.

### 2.9. Antibacterial Activity Assay of Porcine 3D4/31 Lung Alveolar Macrophages

The antibacterial activities of porcine 3D4/31 cells treated with selected HDAC inhibitors were assessed as described [17, 37, 38]. Briefly, 3D4/31 cells were seeded in complete RPMI 1640 medium containing 10% FBS, 1 mM sodium pyruvate, 100 U/ml penicillin, and 100 mg/ml streptomycin at 8 × 10^5^ cells/well in 6-well plates overnight, followed by stimulation in duplicate with or without 16 mM sodium butyrate, 20 *μ*M SAHA, 5 *μ*M HC toxin, or 20 *μ*M apicidin. After 24 h, cells were scraped, washed twice with calcium- and magnesium-free Hank's balanced salt solution, and resuspended in 100 *μ*l water. Cells were then frozen at −80°C for 20 min, thawed, and sonicated for 30 sec, followed by centrifugation at 12,000 × *g* for 10 min at 4°C. Cell supernatants were collected, and 20 *μ*l of the supernatants was incubated with 80 *μ*l of *E. coli* (ATCC 25922) or *S. aureus* (ATCC 43300) at 2.5 × 10^5^ CFU/ml in 20% trypticase soy broth containing 1 mM NaH_2_PO_4_ and 25 mM NaHCO_3_ in a 96-well plate at 37°C. Bacterial turbidity was measured at OD_600_ using SpectraMax M3 (Molecular Devices, Sunnyvale, CA) at 3, 6, and 9 h.

### 2.10. Data Analysis

One-way or two-way analysis of variance (ANOVA), followed by Dunnett's multiple comparison test, was performed for statistical analyses using GraphPad Prism (San Diego, CA). *P* ≤ 0.05 was considered statistically significant.

## 3. Results

### 3.1. Construction and Optimization of a Cell-Based HTS Luciferase Assay

**Figure 1:**
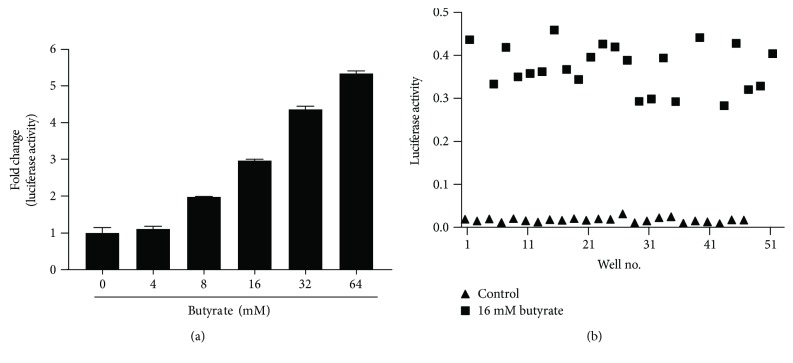
Characterization of a stable porcine IPEC-J2/*PBD3-luc* luciferase reporter cell line: (a) relative fold changes in the relative luciferase activity of the stable cells in response to different concentrations of sodium butyrate for 24 h; (b) relative luciferase activity of the stable cells stimulated with 24 technical replicates in the presence or absence of 16 mM sodium butyrate for 24 h.

Because *PBD3* is among the most responsive porcine HDP genes to butyrate and many other fatty acids and their analogs [20], the *PBD3* gene promoter was selected for the development of a HTS luciferase assay. A 1121 bp *PBD3* gene promoter fragment immediately upstream of the transcriptional start site was cloned into the pGL4.21[*luc2P*/Puro] luciferase reporter vector. The recombinant plasmid was then transfected into porcine IPEC-J2 cells, followed by 2-week puromycin selection to achieve stable integration of the luciferase reporter gene. The newly established luciferase reporter cell line, IPEC-J2/*PBD3-luc*, showed a clear concentration-dependent increase in the luciferase activity to sodium butyrate, with an approximately 5.5-fold increase in response to 64 mM butyrate (Figure 1(a)). To further evaluate the quality of the cell-based HTS assay, we treated the cells with or without 16 mM butyrate in a 96-well plate, assayed for the luciferase activity, and calculated the *Z*′-factor [30] to be 0.56 (Figure 1(b)). The *Z*′-factor in subsequent HTS assays in different plates was calculated to be in the range of 0.56–0.74, which is considered rather robust in a typical HTS assay [30].

### 3.2. High-Throughput Identification of HDP-Inducing Compounds

**Figure 2:**
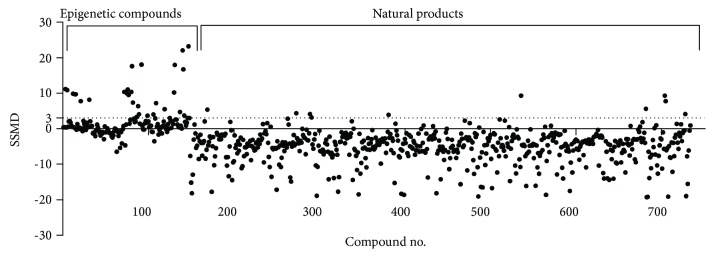
Strictly standardized mean difference (SSMD) values of individual compounds in the primary screening of an Epigenetics Screening Library (compound #1-148) and two natural product libraries (compound #149-732). The IPEC-J2/*PBD3*-*luc* luciferase reporter cell line was stimulated with 20 *μ*M of each compound in the Epigenetics Screening Library or 20 *μ*g/ml of each compound in the natural product libraries for 24 h, followed by the luciferase assay. The alamarBlue Dye was added 4 h before the luciferase assay to measure cell viability. The luciferase activity of each compound was normalized to cell viability before the SSMD value was calculated.

After screening 148 epigenetic and 584 natural compounds, a total of 41 nonredundant compounds showed a SSMD value of no less than 3.0, which are considered to be strong hits [32] (Figure 2). Among these, 33 compounds were from the Epigenetics Screening Library (Table 1), and another 10 compounds were from two natural product libraries (Table 2), with apicidin and *Helminthosporium carbonum* (HC) toxin being identified from both sources. It was apparent that most compounds identified are histone deacetylase (HDAC) inhibitors, whose action leads to hyperacetylation of histones [39] and often increased HDP gene transcription in humans [40]. Additionally, a few histone or DNA methylation inhibitors such as UNC0642 [41], UNC0224 [42], UNC1215 [43], and S-adenosylhomocysteine [44] were also found to have potential HDP-inducing activities (Table 1). Several natural products such as ikarugamycin [45, 46], cynaroside [47], and sanguinarine [48] that are known to have anti-inflammatory, antioxidative, and/or antimicrobial activities but with no confirmed epigenetic modification properties were also identified (Table 2). It is surprising to observe that a number of natural products appeared to inhibit HDP gene expression with a SSMD value of less than −3.0 (Figure 2). The putative inhibitory role of these compounds in HDP gene expression is being verified, but characterizations of the HDP-inducing compounds were the focus of this study.

**Figure 3:**
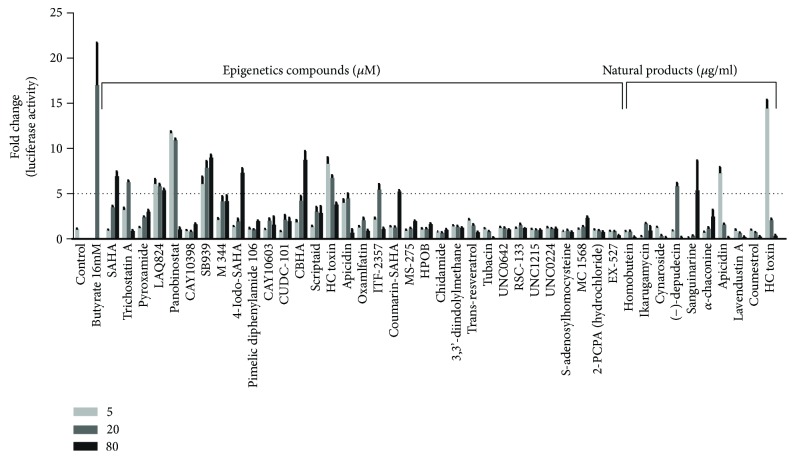
Concentration-dependent changes in the luciferase activity in stable IPEC-J2/*PBD3*-*luc* luciferase reporter cells in response to different concentrations of 43 unique hits identified in the primary screening. For the compounds in the Epigenetics Screen Library, the three concentrations of each compound were 5, 20, and 80 *μ*M, whereas the compounds in the natural product libraries were applied at 5, 20, and 80 *μ*g/ml in duplicate for 24 h. Sodium butyrate at 16 mM was used as the positive control. Note that apicidin and HC toxin were identified from both libraries. The results are means ± SEM of three independent experiments.

Follow-up concentration-response experiments in the stable IPEC-J2/*PBD3-luc* reporter cell line with 41 hits revealed that most compounds increased the luciferase activity in an obvious concentration-dependent manner (Figure 3). A total of 13 nonredundant compounds showed a minimum 5-fold increase in at least one of the three concentrations examined. These consisted of 10 compounds, i.e., trichostatin A (TSA), suberoylanilide hydroxamic acid (SAHA), 4-iodo-SAHA, coumarin-SAHA, m-carboxycinnamic acid bis-hydroxamide (CBHA), panobinostat, HC toxin, ITF-2357, LAQ824, and SB939, from the Epigenetics Screening Library and another four compounds, i.e., (−)-depudecin, sanguinarine, apicidin, and HC toxin, from two natural product libraries (Figure 3). HC toxin was again identified from both sources.

### 3.3. Validation of HDP-Inducing Compounds in Porcine Cell Lines

**Figure 4:**
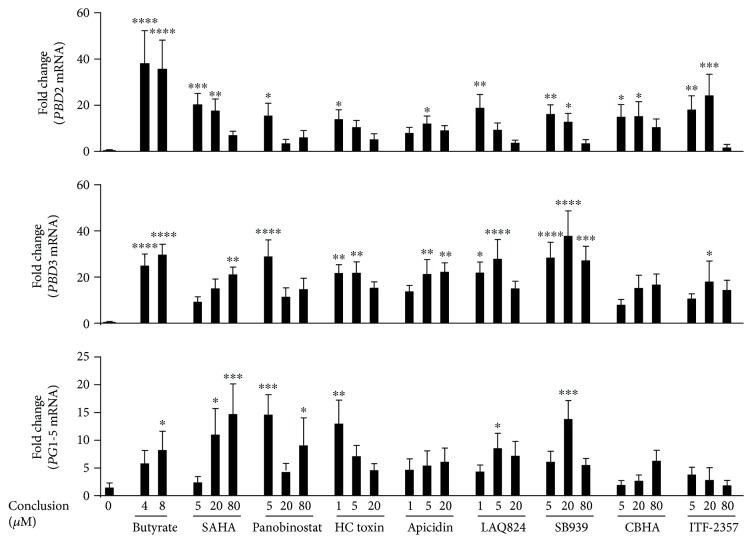
Induction of representative porcine HDP mRNA expression in porcine IPEC-J2 intestinal epithelial cells by the eight selected compounds for 24 h. *PBD2*, *PBD3*, and *PG1–5* mRNA expression levels were quantified using RT-qPCR. Two concentrations (4 and 8 mM) of sodium butyrate were used as positive controls. The results are means ± SEM of three independent experiments. One-way analysis of variance (ANOVA), followed by Dunnett's multiple comparison test, was performed. ^∗^*P* < 0.05, ^∗∗^*P* < 0.01, ^∗∗∗^*P* < 0.001, and ^∗∗∗∗^*P* < 0.0001 (relative to the unstimulated control).

Among the 13 compounds identified, 12 happened to be HDAC inhibitors. Sanguinarine is the only exception; however, it was found to be obviously toxic to IPEC-J2 cells at HDP-inducing concentrations. Another compound, (−)-depudecin, showed no HDP-inducing activity at various concentrations when purchased from BioVision (Milpitas, CA) and MyBioSource (San Diego, CA), which are the only two vendors that we found selling the chemical. Both sanguinarine and (−)-depudecin were, therefore, excluded from further evaluations. We also excluded TSA, a well-known toxic HDAC inhibitor, as well as two SAHA analogs (4-iodo-SAHA and coumarin-SAHA) to minimize redundancy with SAHA. Each of the eight remaining compounds was then assessed at three different concentrations using real-time PCR in the parental IPEC-J2 cell line for their ability to induce the mRNA expression of *PBD3* and other representative porcine HDP genes including *PBD2* and five highly homologous cathelicidins (*PG1–5*), which could not be differentiated by the primers that were used [20, 49]. All eight compounds induced *PBD2* mRNA expression by 10- to 30-fold at a minimum of one concentration examined (Figure 4). They also increased *PBD3* mRNA expression by 15- to 40-fold, while enhancing *PG1–5* expression by 5- to 15-fold (Figure 4). A majority of the eight compounds achieved a similar HDP-inducing efficacy to butyrate. It is also clear that three HDP genes were differentially regulated by these compounds. For example, SAHA concentration dependently increased *PBD3* and *PG1–5* gene expression, but the same three concentrations caused a graduated decrease in *PBD2* gene induction. ITF-2357 was among the most potent compounds in inducing *PBD2* expression but became the least effective in *PBD3* and *PG1–5* gene induction (Figure 4).

**Figure 5:**
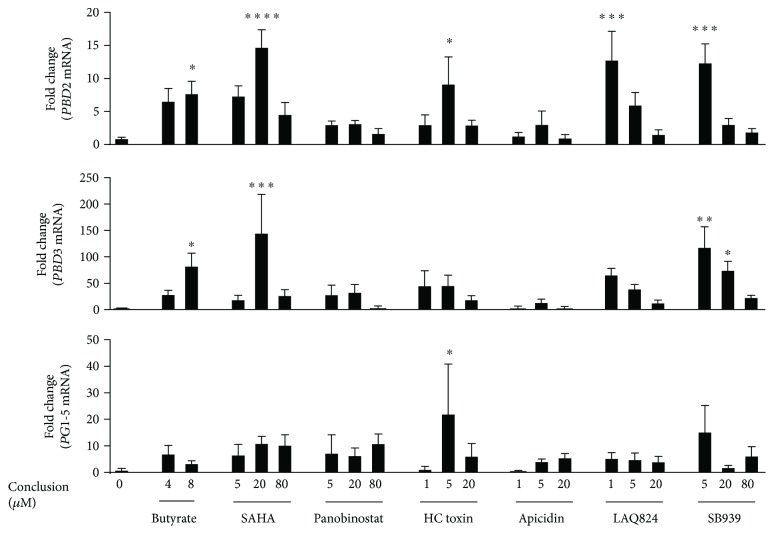
Induction of representative porcine HDP mRNA expression in porcine 3D4/31 lung alveolar macrophage cells by the selected HDAC inhibitors for 24 h. *PBD2*, *PBD3*, and *PG1–5* mRNA expression levels were quantified using RT-qPCR. Two concentrations (4 and 8 mM) of sodium butyrate were used as positive controls. The results are means ± SEM of 2–3 independent experiments. One-way analysis of variance (ANOVA), followed by Dunnett's multiple comparison test, was performed. ^∗^*P* < 0.05, ^∗∗^*P* < 0.01, ^∗∗∗^*P* < 0.001, and ^∗∗∗∗^*P* < 0.0001 (relative to the unstimulated control).

To further compare relative HDP-inducing activities of these compounds in a different cell type, we selected six compounds including SAHA, panobinostat, HC toxin, apicidin, LAQ824, and SB939 that were among the most effective HDP inducers in IPEC-J2 cells and evaluated them in a porcine lung alveolar macrophage cell line, 3D4/31. As expected, all six compounds approached or exceeded the efficacy of butyrate and induced the expression of *PBD2* mRNA by up to 15-fold, *PBD3* by as much as 150-fold, and *PG1–5* by up to 20-fold (Figure 5). Again, we observed differential regulation among HDP genes. For example, LAQ824 and SB939 showed a concentration-dependent decrease in inducing *PBD2* and *PBD3* gene expression; however, such a concentration-response pattern was not obvious with *PG1–5* (Figure 5). Panobinostat at 80 *μ*M appeared to be the least effective in inducing *PBD2* and *PBD3* but was the most effective in inducing *PG1–5*. Nevertheless, it can be concluded that the HTS assay that we developed in this study is highly effective in identifying HDP inducers and that most compounds identified are capable of inducing the expression of multiple porcine HDP genes in different cell types. Enhancing the expression of HDP genes is expected to improve bacterial clearance and disease outcomes [8–10].

### 3.4. No Direct Antibacterial Activity of HDP-Inducing HDAC Inhibitors

To examine whether the HDAC inhibitors identified above have direct antibacterial activities, a representative Gram-negative bacterium (*E. coli*, ATCC 25922) and a Gram-positive bacterium (*S. aureus*, ATCC 43300) were used to determine the minimum inhibitory concentrations of SAHA, apicidin, and HC toxin using a standard broth microdilution assay [33]. However, none of the compounds showed any obvious antibacterial activity, and their minimum inhibitory concentrations were all beyond 320 *μ*M, the highest concentration that we tested (data not shown), implying that these compounds could potentially enhance HDP synthesis and bacterial clearance without exerting selective pressure on bacteria.

### 3.5. Augmentation of the Antibacterial Activity of Porcine 3D4/31 Cells by HDAC Inhibitors

**Figure 6:**
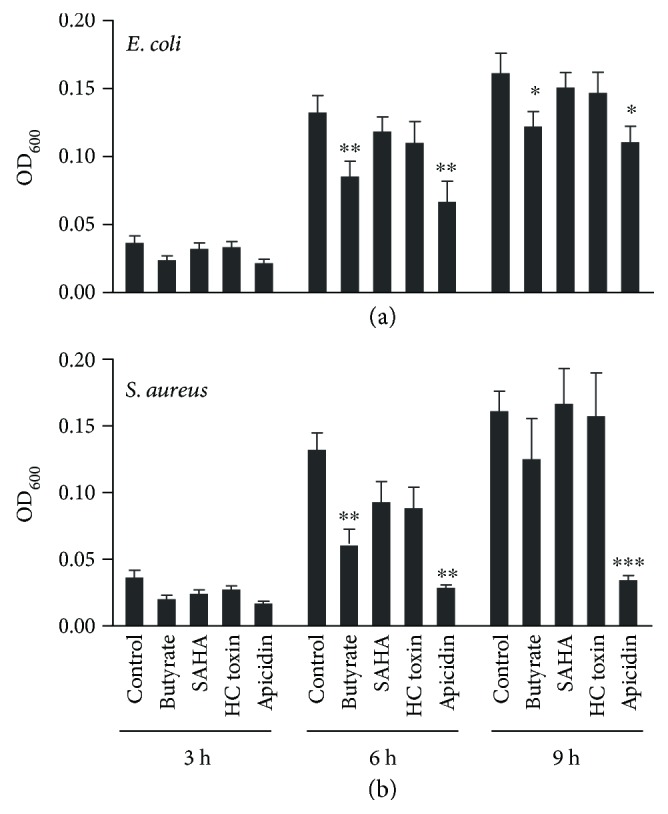
Augmentation of the antibacterial activity of porcine 3D4/31 macrophages by the selected HDAC inhibitors. Porcine 3D4/31 cells were left unstimulated or stimulated with 20 *μ*M SAHA, 20 *μ*M apicidin, or 5 *μ*M HC toxin for 24 h. Sodium butyrate (16 mM) was used as a positive control. Cell lysates were then incubated with *E. coli* ATCC 25922 (a) or *S. aureus* ATCC 43300 (b) at 37°C for 3, 6, and 9 h. The bacterial turbidity was measured at OD_600_. The results are means ± SEM of 2–3 independent experiments. Two-way analysis of variance (ANOVA), followed by Dunnett's multiple comparison test, was performed. ^∗^*P* < 0.05, ^∗∗^*P* < 0.01, and ^∗∗∗^*P* < 0.001 (relative to the control at each time point).

To further confirm whether HDAC inhibitor-mediated HDP mRNA induction can lead to an increase in the antibacterial activity of host cells, porcine 3D4/31 macrophages were left untreated or treated with 16 mM sodium butyrate, 20 *μ*M SAHA, 20 *μ*M apicidin, or 5 *μ*M HC toxin for 24 h, followed by incubation of the cell lysate with *E. coli* (ATCC 25922) or *S. aureus* (ATCC 43300) and measurement of the bacterial turbidity [17, 37, 38]. Consistent with our earlier observation [17], butyrate enhanced the antibacterial activity of 3D4/31 cells (Figure 6). SAHA, apicidin, and HC toxin also similarly improved the ability of porcine macrophages to suppress the growth of both Gram-negative and Gram-positive bacteria, albeit with varying antibacterial efficacies (Figure 6). Further characterization of the efficacy of these compounds in HDP induction and pathogen clearance in live animals could potentially lead to the identification of antibiotic alternatives for use in pigs without triggering bacterial resistance to these HDP-inducing compounds.

## 4. Discussion

A variety of small-molecule compounds have been found to augment HDP synthesis in different animal species [8–10]; however, not all compounds can regulate HDP gene expression with similar efficacy across species [27], which necessitates the identification of species-specific HDP-inducing compounds. A HTS assay was previously developed and led to the discovery of a number of compounds with the ability to induce human cathelicidin *LL-37* [50]. In this study, we developed a cell-based HTS assay to identify HDP inducers for use in pigs. Porcine IPEC-J2 intestinal epithelial cells were permanently integrated with a luciferase reporter vector driven by a *PBD3* gene promoter construct through puromycin selection. After optimization, such a HTS assay had a *Z*′-factor in the range of 0.57–0.74, which suggested that it is a rather robust system and equivalent to a human HTS assay [50]. However, instead of fusing a 4 kb *LL-37* gene promoter plus its entire four-exon open reading frame with the luciferase reporter gene as in the human HTS system [50], our system only needed a 1.1 kb *PBD3* gene promoter (Figure 2) and has resulted in the identification of a number of compounds that are capable of inducing multiple porcine HDPs in both an intestinal epithelial cell line and a macrophage cell line. Nevertheless, it is also possible to employ a longer *PBD3* gene promoter to achieve a better outcome.

The *PBD3* gene promoter was selected to drive the luciferase gene expression in this HTS assay due to its strong responsiveness to a panel of fatty acids and their structural analogs in both porcine IPEC-J2 and 3D4/31 cells [20]. Because several other porcine HDP genes such as *PBD2*, epididymis protein 2 splicing variant C (*EP2C*), and protegrins are also readily induced by fatty acids [20], cloning of those HDP gene promoters into the pGL4.21 luciferase reporter vector is expected to yield a similar outcome to the current HTS assay. Indeed, the compounds identified here using the *PBD3* gene promoter-based system are also capable of triggering the induction of *PBD2* and protegrin genes.

Epigenetic modifications of chromatin such as histone acetylation, histone methylation, and DNA methylation play an important role in regulating gene expression [51]. Histone acetylation is regulated by opposing effects of histone acetyltransferases (HATs) and HDACs [49], whereas histone methylation is controlled by histone methyltransferases (HMTs) and histone demethylases [52]. On the other hand, DNA methylation occurs through the action of DNA methyltransferases (DNMTs), while the enzymes and mechanisms for DNA demethylation are only beginning to be elucidated [53]. HDAC inhibitors suppress the removal of the acetyl groups from acetylated histones resulting in hyperacetylation of histones and increased gene transcription [39]. Consistently, a number of HDAC inhibitors have been identified to be capable of inducing human HDP gene expression [40]. Similarly, HMT and DNMT inhibitors reduce methylation of histones and DNA and often lead to enhanced gene expression [51, 52]. Hypermethylation of DNA and histones has been revealed to suppress human HDP gene expression [54–57].

In this study, a screening of a total of 732 epigenetic and natural product compounds has led to identification of 41 nonredundant compounds with a SSMD value of no less than 3.0. Among them, a great majority are known inhibitors of HDACs, HMTs, and/or DNMTs, which is in contrast with the outcome of an earlier human HTS, where a number of non-HDAC inhibitors were identified [50], which may be due to a difference in the selection of compound libraries. Subsequent validation has resulted in discovery of the 13 most potent HDP inducers that gave a minimum 5-fold increase in the luciferase activity in the stable IPEC-J2/*PBD3*-*luc* reporter cell line. Out of the 13 compounds, 12 are HDAC inhibitors, with sanguinarine being the only exception. Sanguinarine is a benzophenanthridine alkaloid extracted from the bloodroot plant *Sanguinaria canadensis* known to have anti-inflammatory, antioxidative, antiproliferative, and proapoptotic properties [58]. Although its mechanisms of action remain unclear [48], sanguinarine was recently identified as a putative inhibitor of heat shock protein 90 (HSP90) [31], whose activity has been demonstrated to be regulated by reversible acetylation [59].

HDACs are classified into four classes (I–IV) based on their sequence homology and domain organization, while HDAC inhibitors are often grouped according to their structures [49]. Both broad-spectrum and class-specific HDAC inhibitors exist [49]. Among the 12 HDAC inhibitors identified, most are known pan-HDAC inhibitors that are capable of suppressing the activity of multiple classes of HDACs and the majority are hydroxamates (Table 3). For example, SAHA is a well-known pan-HDAC inhibitor that has been approved by the FDA in 2006 for treatment of advanced primary cutaneous T-cell lymphoma [60]. In this study, we found that SAHA is highly active in inducing multiple porcine HDP gene expression in both IPEC-J2 and 3D4/31 cells. HC toxin, a cyclic tetrapeptide first isolated from the secondary metabolite of *Helminthosporium carbonum* [61], is also highly efficient in enhancing HDP expression in both porcine intestinal epithelial and alveolar macrophages. Similarly, SB939, also known as pracinostat, is a synthetic pan-HDAC inhibitor [62, 63] and effective in HDP induction in both cell types as well.

Interestingly, MS-275, also known as entinostat, was identified in humans to be among the most effective HDAC inhibitors in *LL-37* and *β*-defensin 1 (*HBD1*) gene expression [64, 65]; however, it failed to give more than 5-fold increase in the luciferase activity in follow-up evaluations in the stable IPEC-J2/*PBD3-luc* luciferase reporter cell line in our study, albeit with a SSMD value of 7.39 at 20 *μ*M in the initial screening (Table 1), suggesting a difference in the potency of various HDP-inducing compounds among animal species. In fact, species-specific HDP induction by HDP inducers has been observed. Vitamin D_3_, which is a potent human cathelicidin *LL-37* inducer, completely loses its ability to induce the cathelicidin gene expression in mice [27]. Medium-chain fatty acids such as hexanoate (C6) and heptanoate (C7) are more potent than short-chain fatty acids such as butyrate (C4) and valeric acid (C5) in HDP induction in human cells [19], while the opposite is true in chickens and pigs [18, 20].

Besides showing a species-specific regulation pattern, many HDP genes are regulated in cell- and gene-specific manners as well. First, a HDP gene is often modulated differently by the same inducer in different cell types. For example, human *LL-37* is strongly induced by butyrate in intestinal epithelial cells but with a minimum change in mRNA expression in response to butyrate in monocytes or skin keratinocytes [37]. In this study, *PBD2*, *PBD3*, and *PG1–5* are induced to different magnitudes by many of the same compounds in porcine IPEC-J2 epithelial and 3D4/31 macrophages. Secondly, different HDP genes are often regulated differently in a cell type by the same inducer. Human *LL-37* is readily induced by vitamin D_3_ in monocytes and keratinocytes, where *HBD1* and *HBD2* are barely altered by vitamin D_3_ [37]. Here, we observed that 20 *μ*M SAHA increased *PBD3* expression by nearly 150-fold in 3D4/31 cells but only enhanced *PBD2* or *PG1–5* expression by 10- to 15-fold (Figure 5). It is, therefore, desirable to identify HDP inducers with the capacity to induce the synthesis of multiple HDPs in multiple cell types across animal species. To date, most HDAC inhibitors appear to have this capacity by simultaneously inducing multiple HDP synthesis in different cell types and different animal species, albeit with varying potency [40]. Besides their use in swine, the HDAC inhibitors identified in this study could thus see potential application in other animals.

## 5. Conclusions

We have successfully developed a cell-based HTS assay and identified several highly active HDP inducers with the potential for further development as antibiotic alternatives for swine use. Because most HDAC inhibitors work across species, these small-molecule compounds are likely to be able to induce HDP synthesis and enhance disease resistance in other animals and even humans. Therapeutic and prophylactic applications of those HDP-inducing compounds with no direct antimicrobial activities constitute a novel host-directed approach to infectious disease control and prevention with virtually no risk of triggering microbial resistance.

## Figures and Tables

**Table 1:** Strictly standardized mean difference (SSMD) values and major functions of 33 hits at 20 *μ*M from the primary screening of the Epigenetics Screening Library.

Compound name	CAS number	SSMD	Major function
SAHA	149647-78-9	23.22	HDAC inhibitor
Trichostatin A	58880-19-6	22.09	HDAC inhibitor
Pyroxamide	382180-17-8	18.1	HDAC inhibitor
LAQ824	404951-53-7	17.98	HDAC inhibitor
Panobinostat	404950-80-7	17.61	HDAC inhibitor
CAY10398	193551-00-7	16.72	HDAC inhibitor
SB939	929016-96-6	11.16	HDAC inhibitor
M 344	251456-60-7	11.07	HDAC inhibitor
4-Iodo-SAHA	1219807-87-0	10.86	HDAC inhibitor
Pimelic diphenylamide 106	937039-45-7	10.38	HDAC inhibitor
CAY10603	1045792-66-2	10.36	HDAC inhibitor
CUDC-101	1012054-59-9	10.23	HDAC/EGFR/HER2 inhibitor
CBHA	174664-65-4	9.9	HDAC inhibitor
Scriptaid	287383-59-9	9.87	HDAC inhibitor
HC toxin	83209-65-8	9.72	HDAC inhibitor
Apicidin	183506-66-3	9.71	HDAC inhibitor
Oxamflatin	151720-43-3	9.54	HDAC inhibitor
ITF-2357	732302-99-7	8.19	HDAC inhibitor
Coumarin-SAHA	1260635-77-5	7.78	HDAC inhibitor
MS-275	209783-80-2	7.39	HDAC inhibitor
HPOB	1429651-50-2	7.2	HDAC inhibitor
Chidamide	743420-02-2	6.38	HDAC inhibitor
3,3′-Diindolylmethane	1968-05-4	5.52	HDAC/DNMT inhibitor
*trans*-Resveratrol	501-36-0	4.71	HDAC inhibitor
Tubacin	537049-40-4	4.06	HDAC inhibitor
UNC0642	1481677-78-4	4.04	HMT inhibitor
RSC-133	1418131-46-0	3.72	HDAC/DNMT inhibitor
UNC1215	1415800-43-9	3.67	L3MBTL3 inhibitor
UNC0224	1197196-48-7	3.39	HMT inhibitor
S-Adenosylhomocysteine	979-92-0	3.21	DNMT inhibitor
MC 1568	852475-26-4	3.05	HDAC inhibitor
2-PCPA (hydrochloride)	1986-47-6	3.02	LSD1 inhibitor
EX-527	49843-98-3	3.0	HDAC inhibitor

Abbreviations: HDAC: histone deacetylase; EGFR: epidermal growth factor receptor; HER2: human epidermal growth factor receptor 2; DNMT: DNA methyltransferase; HMT: histone methyltransferase; L3MBTL3, lethal (3) malignant brain tumor-like protein 3, a histone methyl-lysine binding protein; LSD1: histone lysine-specific demethylase 1.

**Table 2:** Strictly standardized mean difference (SSMD) values and major functions of 10 hits at 20 *μ*g/ml from the primary screening of two natural product libraries (BIOMOL International).

Compound name	CAS number	SSMD	Major epigenetic function
Homobutein	34000-39-0	9.32	HDAC inhibitor
Ikarugamycin	36531-78-9	9.3	Unknown
Cynaroside	5373-11-5	7.79	Unknown
(−)-Depudecin	139508-73-9	5.63	HDAC inhibitor
Sanguinarine	2447-54-3	5.36	Unknown
*α*-Chaconine	20562-03-2	4.36	Unknown
Apicidin	183506-66-3	4.13	HDAC inhibitor
Lavendustin A	125697-92-9	4.11	Unknown
Coumestrol	479-13-0	3.91	Unknown
HC toxin	83209-65-8	3.16	HDAC inhibitor

**Table 3:** Chemical structures and classification of 13 compounds giving a minimum of 5-fold increase in the luciferase activity in stable porcine IPEC-J2/*PBD3*-*luc* luciferase reporter cells.

Chemical name	Chemical structure	Class of HDAC inhibitor	Types of HDAC inhibited
SAHA	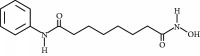	Hydroxamate	Class I, II, and IV HDACs
4-Iodo-SAHA	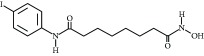	Hydroxamate	Class I, II, and IV HDACs
Coumarin-SAHA	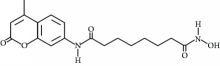	Hydroxamate	Class I, II, and IV HDACs
Trichostatin A	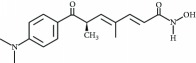	Hydroxamate	Class I, II, and IV HDACs
LAQ824	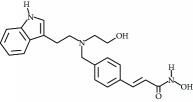	Hydroxamate	Class I and II HDACs
Panobinostat	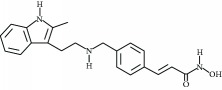	Hydroxamate	Class I, II, and IV HDACs
SB939	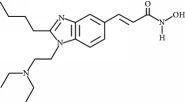	Hydroxamate	Class I, II, and IV HDACs
CBHA	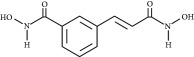	Hydroxamate	Class I and II HDACs
ITF-2357	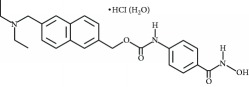	Hydroxamate	Class I and II HDACs
HC toxin	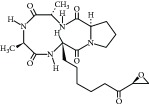	Cyclic tetrapeptide	Class I and II HDACs
Apicidin	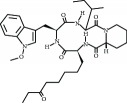	Cyclic tetrapeptide	Class I and II HDACs
(−)-Depudecin	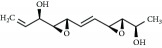	Epoxide	Class I HDACs
Sanguinarine	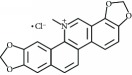	N/A	N/A

## Data Availability

The data used to support the findings of this study are available from the corresponding author upon request.
